# Rational Design of a Chimeric Derivative of PcrV as a Subunit Vaccine Against *Pseudomonas aeruginosa*

**DOI:** 10.3389/fimmu.2019.00781

**Published:** 2019-04-24

**Authors:** Chuang Wan, Jin Zhang, Liqun Zhao, Xin Cheng, Chen Gao, Ying Wang, Wanting Xu, Quanming Zou, Jiang Gu

**Affiliations:** ^1^Department of Microbiology and Biochemical Pharmacy, National Engineering Research Center of Immunological Products, College of Pharmacy, Third Military Medical University, Chongqing, China; ^2^Department of Critical Care Medicine, Children's Hospital of Chongqing Medical University, Chongqing, China

**Keywords:** rational design, vaccine, PcrV, *Pseudomonas aeruginosa*, pneumonia

## Abstract

*Pseudomonas aeruginosa* (PA) is a major cause of nosocomial infections, which remain an unsolved problem in the clinic despite conventional antibiotic treatment. A PA vaccine could be both an effective and economical strategy to address this issue. Many studies have shown that PcrV, a structural protein of the type 3 secretion system (T3SS) from PA, is an ideal target for immune prevention and therapy. However, difficulties in the production of high-quality PcrV likely hinder its further application in the vaccine industry. Thus, we hypothesized that an optimized PcrV derivative with a rational design could be produced. In this study, the full-length PcrV was divided into four domains with the guidance of its structure, and the Nter domain (Met1-Lys127) and H12 domain (Leu251-Ile294) were found to be immunodominant. Subsequently, Nter and H12 were combined with a flexible linker to generate an artificial PcrV derivative (PcrV_NH_). PcrV_NH_ was successfully produced in *E. coli* and behaved as a homogenous monomer. Moreover, immunization with PcrV_NH_ elicited a multifactorial immune response and conferred broad protection in an acute PA pneumonia model and was equally effective to full-length PcrV. In addition, passive immunization with anti-PcrV_NH_ antibodies alone also showed significant protection, at least based on inhibition of the T3SS and mediation of opsonophagocytic killing activities. These results provide an additional example for the rational design of antigens and suggest that PcrV_NH_ is a promising vaccine candidate for the control of PA infection.

## Introduction

*Pseudomonas aeruginosa* (PA) is a major cause of nosocomial infections in patients with compromised immunity (1). In patients with impaired respiratory functions, PA-induced pneumonia contributes to a larger proportion of mortality, such as from cystic fibrosis (2), long-term mechanical ventilation (3), and COPD (4). With increased drug resistance, the effectiveness of antibiotic therapies is limited. As a result, it remains difficult to combat PA infection despite supportive treatments. Vaccines could be an alternative strategy to control PA infections and even reduce antibiotic resistance; however, no PA vaccine is currently available (5).

PA possesses a type III secretion system (T3SS) that directly injects toxic effectors (ExoU, ExoS, ExoY, ExoT) into the host cells and causes subsequent tissue damage (6). The T3SS apparatus consists of three parts: a basal complex spanning the bacterial membrane, an external needle, and a pore-forming component. The PA V-antigen (PcrV) forms a ring structure at the tip of the needle and is crucial for translocation of the effectors (7). Deletion of the *pcrV* gene results in nearly complete abolition of pathogenicity by disrupting the delivery of toxic effectors (8, 9). In addition, PcrV contributes to the proper assembly of the pore-forming translocon PopB/PopD (10).

Antibodies targeting PcrV have been shown to be effective in combating PA infection in multiple animal models (11) and even cystic fibrosis patients (12). Additionally, data from a phase 2 trial of a recombinant anti-PcrV Fab fragment (KB001) showed that it could reduce inflammation and damage of the airway of CF patients (13). The phase 1 clinical trial of a bispecific antibody (MEDI3902) targeting PcrV and Psl was recently completed (NCT02255760) (14). Moreover, two studies founded that polyclonal anti-PcrV IgGs derived from human sera conferred protection against PA caused pneumonia in mice (15, 16). Nevertheless, a limited number of reports has focused on the active immunization of PcrV. Vaccination with recombinant PcrV DNA elicited protective immunity against acute pneumonia and decreased lung inflammation and damage (17–19). A recent study showed that nasal immunization with PcrV adjuvanted with CpG was able to elicit PcrV specific IgA and IgG antibodies and provided protection against PA pneumonia (20). One possible reason for the restricted application of PcrV for vaccine development is the difficult production of high-quality protein, as we observed in our previous studies (21).

With the aid of protein structure tools, an increasing number of antigens has been designed or optimized to obtain better performance (22). For example, the membrane-anchored fusion glycoprotein F of Respiratory Syncytial Virus (RSV) was a target for therapeutic antibodies; however, it tends to aggregate by a hydrophobic fusion peptide, which hinders its development as a vaccine candidate. By structure-based design, a highly stable form of RSV F antigen was generated via removal of the fusion peptide. The mutated F antigen molecule was found to induce effective neutralizing antibodies in multiple animal models and advanced to the clinical trial phase (23). Thus, we hypothesized that the structure tools could be used to resolve the difficulties in PcrV production. In this study, the immunogenicity of the four domains of PcrV was determined with the guidance of its structure. An artificial PcrV derivative (PcrV_NH_), which consisted of its two immune dominant domains, was successfully produced and tested as a vaccine candidate in mice.

## Materials and Methods

### Animals, Strains, and Sera

Six- to Eight-week-old specific pathogen-free female BALB/c mice were purchased from Beijing HFK Bioscience Limited Company (Beijing, People's Republic of China). PA XN-1(CCTCC M2015730) was isolated from a severe pneumonia patient in Southwest Hospital in China. Strain PA103 (ATCC 29260) was purchased from ATCC. Clinical strains PA ZJ-01, PA GZ-18, PA BJ-15, and PA KM-9 were isolated from Zhejiang, Guangzhou, Beijing, and Kunming City of China. The serotypes of these stains were determined by Mei serotyping kit (Mei assay, Meiji Seika). Rabbits were purchased from the animal experimental center of Third Military Medical University.

Sera were collected from PA-infected patients and healthy donors in Southwest Hospital in China. Written informed consent was collected. All animal care and experiments in this study abided by the ethical regulations and were approved by the Animal Ethical and Experimental Committee of the Third Military Medical University (NO. TMMU0159).

### Production of PcrV and Its Derivatives

The structure model of PcrV was built up by I-TASSER Suite (24). Full-length PcrV and predicted subdomains fused with MBP (maltose-binding protein) were constructed. In brief, the nucleotide sequences encoding these constructs were synthesized and cloned into the pMAL-c5X vector (NEB) using BamHI and EcoRI restriction sites. The sequence of recombinants was confirmed by DNA sequencing. These recombinants were transformed into *E. coli* XL-Blue-1 for production of recombinant proteins.

The expression of recombinant proteins was induced by adding 0.2 mM (final concentration) IPTG to 1 L of cell culture in LB medium when the OD_600_ reached 0.5. The cell cultures were grown at 16°C for an additional 15 h. Cells were then collected by centrifuge at 5,000 rpm for 10 min and lysed by sonication in lysis buffer (20 mM PB, pH 7.0, 150 mM NaCl) on ice. The cell lysate was centrifuged at 12,000 rpm for 15 min, and the supernatant was then collected and passed through a column of maltose resin (NEB). After washing with 5-fold column volumes of washing buffer (20 mM PB, pH 7.0, 1 M NaCl) three times, 50 mM maltose was added to the column to elute the target proteins. The eluted proteins finally were concentrated in buffer (20 mM PB, pH 7.0, 150 mM NaCl) and identified by SDS-PAGE. Finally, proteins were quantified by BCA assay and stored at −80°C for future assays.

For production of PcrV_NH_, the gene encoding PcrV_NH_ was synthesized and cloned into pGEX-6p-1(GE Healthcare) by BamHI and Xhol restriction sites. The induction of PcrV_NH_ was the same as that for full-length PcrV fused with MBP. To purify PcrV_NH_, the induced cells were collected and lysed by sonication in lysis buffer (20 mM Tris-HCl, pH 8.0, 200 mM NaCl). The cell lysate was centrifuged at 12,000 rpm for 15 min, and the supernatant was then collected and passed through a glutathione resin column (NEB). After washing with 5-fold column volumes of washing buffer (20 mM Tris-HCl, pH 8.0, 1.0 M NaCl) three times, PreScission protease (GE Healthcare) was added and incubated at 4°C for 12 h. PcrV_NH_ was then eluted from the glutathione resin and passed through a Resource Q column (GE Healthcare) in buffer (20 mM Tris-HCl, pH 8.0, 100 mM NaCl) and eluted with a gradient (0–100%, 10 column volumes) of elution buffer (20 mM Tris-HCl, pH 8.0, 1.0 M NaCl). The eluted PcrV_NH_ was finally concentrated in buffer (20 mM Tris-HCl, pH 8.0, 100 mM NaCl) and stored at −80°C for future assays.

### ELISA

The response of full-length PcrV and subdomains to serum from PA-infected patients was measured by standard ELISA. Briefly, microplates were coated with 0.2 μg of MBP tagged PcrV, Nter, H7, H12, or Cter per well. Free MBP protein was used as a control. Sera were then added at a dilution of 1:100 and incubated at 37°C for 1 h. After washing the microplates three times, HRP (horseradish peroxidase)-labeled goat anti-human Fc antibodies (1:5,000 diluted, Abcam) were added and incubated at 37°C for 40 min. Then, TMB substrate was added and incubated at 37°C for 10 min to develop the color. Sulfuric acid (2.0 M) was introduced to terminate the reaction; the optical density was measured at 450 nm (OD_450_), and the percentage of OD to PcrV was calculated by the following formula: Subdomain% = (OD_subdomain_-OD_MBP_)/(OD_PcrV_-OD_MBP_)^*^100%.

### Assay of ExoU Secretion *in vitro*

To produce anti-Nter, -H7, -H12, and -Cter polyclonal antibodies, 20 mice were equally divided into four groups. Each group was intramuscularly immunized with 30 μg of Nter, H7, H12, or Cter at 0, 14, and 21 days. Serum from immunized mice was obtained 2 weeks after the final immunization. PA103 was grown in LB medium mixed with PBS, anti-Nter, -H7, -H12, or -Cter antibodies (1.0 mg/ml). After incubation for 18 h, the amount of PA103 was determined by measuring the OD_600_ and standardized to ensure that same number of bacteria was analyzed. In addition, the bacteria pellets were collected and subject to SDS-PAGE. Then the content of flagellin was determine by Western Blot and used as a control. Meanwhile, ExoU in the supernatant was collected and concentrated as described previously (25). The quantity of ExoU was analyzed by Western blot using anti-ExoU antibodies(self-developed) as primary antibodies. Additionally, a gradient concentration ranging from 10 to 0.31 mg/ml of anti-PcrV_NH_ antibodies was used to determine their effects on the secretion of ExoU. Moreover, the effects of antibodies on the secretion of ExoU were evaluated under a T3SS inducing condition. To date, PA103 was grown in Trypticase soy broth with 10 mM nitrilotriacetic acid as previously described (26), the rest procedures were same as that for bacteria grown in LB medium.

### Determination of the Bacterial Cytotoxicity *in vitro*

The effects of anti -PcrV fragments and -PcrV_NH_ antibodies on the bacterial cytotoxicity were evaluated in Hela cells as described before (27). In brief, Hela cells (1 × 10^5^ per well) were cultured in DMEM supplemented with 10% fetal calf serum at 37°C with 5% CO_2_ for 24 h. Three hours before infection, cells were washed with sterilized PBS for three times and incubated in fresh DMEM with 10% fetal calf serum. PA103 after overnight culture was subcultured to the log phase (OD_600_ = 0.6–0.8) in fresh LB. The bacteria were then collected by centrifuge and washed with sterilized PBS for three times. These bacteria were then added into Hela cells at multiplicity of infection (MOI) of 20. Meanwhile, anti-PcrV fragments or different concentrations of anti-PcrV_NH_ antibodies were introduced. Three hours post infection, the culture medium in each well was removed. After washing with PBS for three times, the attached cells were stained with 0.01% crystal violet for 15 min at 37°C. The plates were then washed with water for three times. Finally, 95% ethanol was added to each well to dissolved the Hela cell-associated crystal violet dye, and the optical density was measured at 590 nm.

### Characterization of the Oligomerization and Homogeneity of PcrV_NH_

First, the oligomerization of purified PcrV_NH_ was analyzed by size-exclusion chromatography as previously described with few modifications (28). The protein standards Blue dextran 2000, aldolase, conalbumin, ovalbumin, carbonic anhydrase (CA), and ribonuclease A (R) were purchased from GE Healthcare. PcrV_NH_ and the protein standards were diluted to 5 mg/ml and loaded onto a Superdex 200 16/60 column in buffer (20 mM PB, pH 7.0, 150 mM NaCl). The elution volume of peaks of PcrV_NH_ and the protein standards were determined. The predicted Mw of PcrV_NH_ was calculated as described by Wang et al. (29). Second, the dynamic light-scattering assay was applied to characterize PcrV_NH_ in the water phase. In brief, purified PcrV_NH_ was diluted to 0.5 mg/mL and loaded onto a Zetasizer (Malvern, UK) equipped with an argon ion laser. The analysis was then performed three times at 25°C.

### Characterization of PcrV_NH_-Elicited Immunity

To evaluate the immune response, ten mice were equally divided into two groups, which were then immunized intramuscularly with 500 μl of an emulsion of 30 μg of purified PcrV_NH_ protein formulated with 500 μg of Al(OH)3 at 0, 14 and 21 days. The other mice were intramuscularly injected with sterile PBS as a control group. Sera were collected 7 days after the last immunization.

The titers of anti-PcrV_NH_, -MBP-Nter, -MBP-Cter, and -MBP-PcrV antibodies were determined by ELISA. In brief, purified PcrV_NH_, MBP-Nter, MBP-Cter, and MBP-PcrV were coated onto 96-well microplates. Serially diluted (2-fold) sera were then added to each well and incubated for 60 min at 37°C. After washing three times with PBST, goat anti-mouse IgG Fc antibodies labeled with HRP (Abcam) were added to the wells and incubated for 40 min at 37°C. Color was developed, and the OD_450_ was determined as described previously. Additionally, the subtype of anti-PcrV_NH_ IgG was analyzed using goat anti-mouse IgG1, IgG2a, and IgG2b mAbs as secondary antibodies by ELISA.

To characterize the cellular immune response, spleens from PcrV_NH_-immunized mice were collected 7 days after the last immunization and homogenized in sterile PBS. After the removal of red blood cells, splenocytes were seeded into 96-well plates (1 × 10^6^ per well) and cultured in DMEM containing 10% FBS. PcrV_NH_ was added into 96-well plates to reach a final concentration of 10 μg/ml and incubated for 36 h to stimulate the splenocytes. Additionally, cells incubated with anti-CD28 and -CD3 antibodies (Biolegend) served as the positive controls. The supernatant was collected to measure the concentrations of IL-4, IFN-gamma, and IL-17A via ELISA according to the manufacturer's instructions (R&D system, Minneapolis, US and Dakewe, ShenZhen, China).

### Immunization and Challenge of Mice

For lethal acute pneumonia challenge, 50 mice were equally divided into five groups, which were then immunized intramuscularly with PcrV_NH_ or MBP-PcrV protein. As control groups, the rest of the mice were intramuscularly injected with sterile PBS, Al(OH)3, and MBP at the same intervals. Afterwards, the mice in each group were anesthetized with pentobarbital sodium followed by the intratracheal injection of a lethal dose of bacteria. The lethal amounts of PA XN-1 were 1.0 × 10^7^. The number of deaths in lethal doses was recorded every 12 h over 7 days.

In addition, ten mice in the immunization and control groups were infected with a sublethal dose to investigate the protection mechanism. The health and weight of mice in the immunization and control groups challenged with a sublethal dose were observed and assessed by breathing, piloerection, movement, nasal secretion, posture, and weight. Then, the global score was recorded as unaffected (0–1), slightly affected (2–4), moderately affected (5–7), or severely affected (8–10).

Furthermore, to evaluate the broad protection of PcrV_NH_-elicited immunity, 40 immunized mice were divided equally into four groups. Seven days after the last immunization, ten mice in each group were challenged by intratracheal injection with four clinical strains: PA ZJ-01 (3.0 × 10^7^ CFU), PA GZ-18 (2.0 × 10^7^ CFU), PA BJ-15 (2.0 × 10^7^ CFU), or PA KM-9 (4.0 × 10^6^ CFU). The survival rates were monitored every 12 h for 7 days.

### Histological Analysis

The lung tissues of mice challenged with a sublethal dose were collected 24 h post-infection. Lung samples were fixed in neutral 10% formalin, embedded in paraffin, sectioned, and stained with hematoxylin and eosin. The sections were observed at 100-fold magnification. Each lung section received a score of 0–4 (from no abnormalities to severe) by a criteria based on hemorrhage, edema, hyperemia, and neutrophil infiltration.

### Quantification of Inflammation in the Infected Lung

To quantify neutrophil infiltration, the bronchoalveolar lavage fluid (BALF) from immunized mice 24 h post-challenge was collected and centrifuged. The pellet was stained using the following antibodies: PE/Cy7 anti-mouse CD45 and APC/Cy7 anti-mouse Ly-6G (Biolegend). Then, the samples were analyzed using BD FACSArray software TM on a BD FACS Array flow cytometer (BD Biosciences). In addition, the concentrations of pro-inflammatory cytokines in the supernatant, such as IL-1β and TNF-α, were quantified by a Mouse Quantikine ELISA. The protocol was performed based on the manufacturer's (Biolegend) instructions.

### Determination of Bacterial Burden

Lung and spleen tissues from mice challenged with a sublethal dose in the immunization and control groups were collected, weighed, and homogenized in 1 ml of sterilized PBS 24 h post-challenge. All homogenates were then plated on LB plates and cultured at 37°C overnight. Numbers of CFUs per gram of tissue were calculated from each plate.

### Evaluation of PcrV_NH_-Specific Antibodies

Rabbits were immunized with 2 ml (0.5 mg/ml) of PcrV_NH_ formulated with 2 ml Freund's adjuvant (Sigma) at 0, 14, and 21 days. Seven days after the final immunization, antibodies in the sera were collected and purified. To evaluate protective efficacy of PcrV_NH_-specific antibodies *in vivo*, 30 mice were equally divided into three groups. Then, each group was intraperitoneally injected with 1.0 mg of anti-PcrV_NH_ antibodies, non-specific rabbit IgGs or PBS. Four hours later, all mice were challenged with a lethal dose of PA XN-1, and survival was observed every 12 h for 7 days.

### Opsonophagocytic Killing Assay

HL-60 cells (ATCC, CCL-240) were differentiated into granulocyte-like cells in growth medium containing 100 mM N′N-dimethylformamide for 5 days. Serum samples from PcrV_NH_ immunized rabbits were heat-inactivated (56°C, 30 min) and serially diluted with opsonization buffer (mix of 80 ml of sterile water, 10 ml of 10 × Hank's balanced salt solution, 10 ml of 1% gelatin, and 5.3 ml of fetal bovine serum). In 96-well plates, each well received 40 μl of 4 × 10^5^ HL-60 cells, 10^3^ CFUs of PA XN-1 in 10 μl of opsonophagocytic buffer, 20 μl serum, and 10 μl of 1% infant rabbit serum as a complement source. After incubation for 2 h, 10 μl of each sample was plated onto agar medium. The opsonophagocytic killing effect was defined as a reduction in CFUs after overnight incubation compared with the CFUs in the sera from unimmunized rabbits.

### Statistical Analysis

The data were presented as the means ± SE. The significance of differences was determined by unpaired parametric test (Student's *t*-test for two groups or one-way ANOVA for more than three groups). Bacterial burden was analyzed by the non-parametric Mann-Whitney test. The survival rate was analyzed by Kaplan-Meier survival curves. SPSS15.0 and GraphPad Prism 6.0 was used for data analysis. Significance was accepted when *P* < 0.05.

## Results

### The Nter and the H12 Are Immunodominant Domains of PcrV

**Figure 1:**
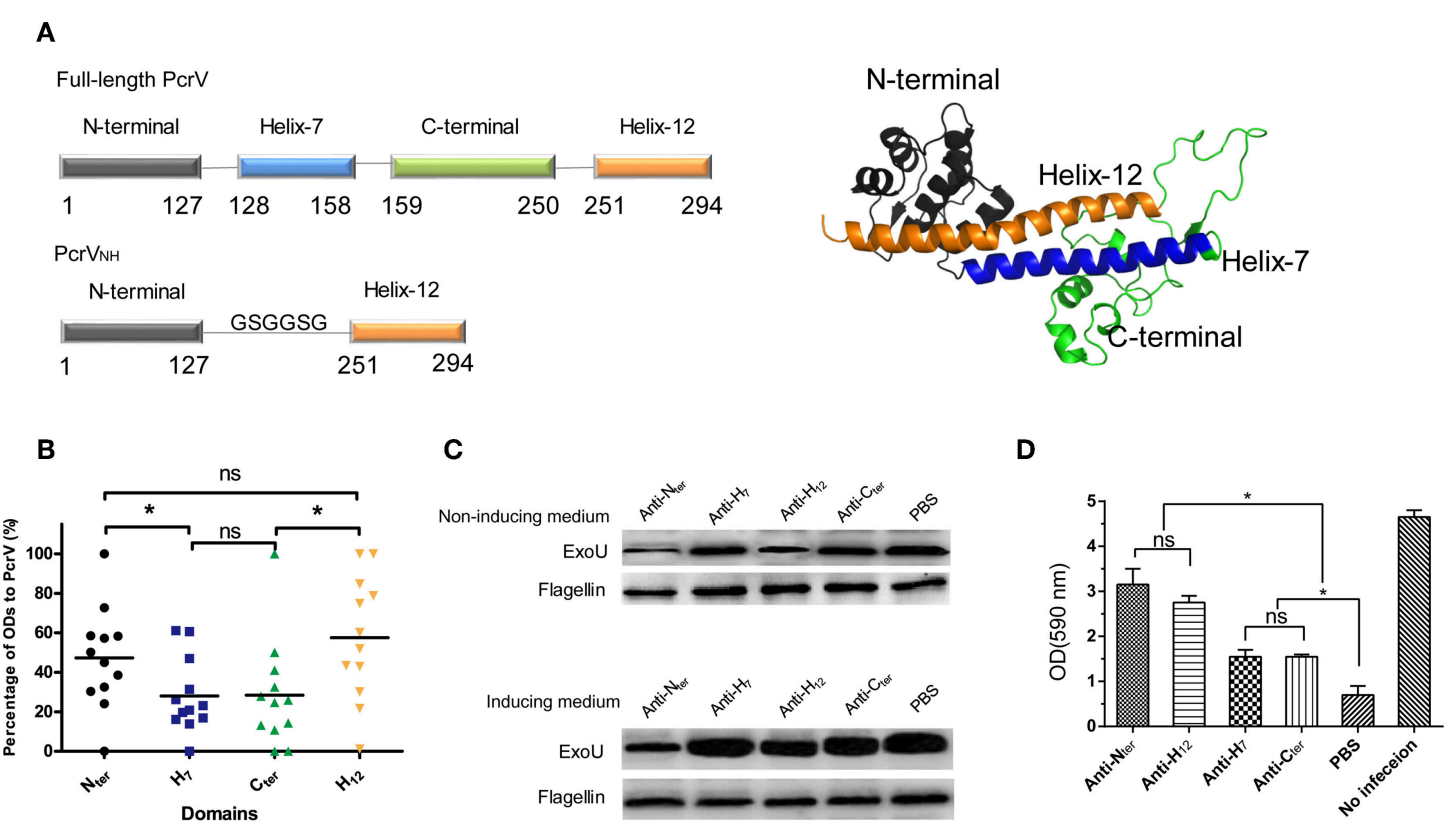
Characterization of the immunogenicities of PcrV subdomains. **(A)** Schematic illustration of the structure of full-length PcrV and PcrV_NH_. The left panel shows that the full-length PcrV was composed of four domains, namely, Nter (Met1-Lys127), H7 (Arg128-Ala158), Cter (Lys159-Pro250), and H12 (Leu251-Ile294). The Nter domain was connected to H12 with a GSGGSG linker to generate ParV_NH_. The cartoon image of the 3D structure of PcrV, predicted from I-TASSER Suite, is shown in the right panel. Nter, H7, Cter, and H12 are colored in gray, blue, green, and orange, respectively. **(B)** The immune response of the subdomain of PcrV to sera from PA-infected patients. The bar represents the relative percentage of OD of each domain to that of full-length PcrV. The immune reactivity of the Nter and H12 domains was significantly stronger than that of the H7 and Cter domains, (*P < 0.05, ns, not significant *P* > 0.05). **(C)** The effect of anti-PcrV subdomain antibodies on the secretion of ExoU. PA 103 grown in ExoU-inducing or non-inducing medium were coincubated with anti-Nter, -H7, -Cter, and -H12 antibodies, and the ExoU in in the culture supernatant was detected by Western blot. The content of flagellin in cell pellets was used as control. Treatment of the anti-Nter and anti-H12 domain antibodies led to a marked reduction of the secretion of ExoU when grown in both the two media. **(D)** The effect of anti-PcrV subdomain antibodies on the T3SS mediated cytotoxicity. Hela cells were infected with PA103 for 3 h and anti-PcrV subdomain antibodies was added into the culture media. The attached cells were stained crystal violet, which was then dissolved in ethanol. The bar represents the optical density at 590 nm of dissolved crystal violet. The data are shown as the means ± SE. *P < 0.05, ns, not significant (*P* > 0.05).

First, the immune reactivity of each domain of PcrV was characterized with the guidance of its structural information. As the atomic structure of PcrV was unavailable, the model of PcrV was built according to its homology with LcrV (PDB: 1R6F) by I-Tasser (24). As shown in Figure 1A, the dumbbell shaped full-length PcrV comprised four domains: Nter (Met1-Lys127), H7 (Arg128-Ala158), Cter (Lys159-Pro250), and H12 (Leu251-Ile294). Then, recombinant MBP-tagged Nter, H7, H12 and Cter were purified, and their reactivity was determined by ELISA using serum from PA-infected patients. MBP and MBP-PcrV_full−length_ were used as negative and positive controls, respectively. To analyze the differences in immune response, the ODs of Nter, H7, H12 and Cter to the serum samples were standardized. Notably, the immune reactivity of Nter and H12 domains was significantly stronger than that of the H7 and Cter domains (*P* < 0.05) (Figure 1B). No significant differences were observed between the Nter and H12 domains (*P* > 0.05) or between the H7 and the Cter domains (*P* > 0.05).

Next, the Nter-, H7-, H12-, and Cter-specific polyclonal antibodies were produced, and their abilities to block the secretion of ExoU mediated by PcrV was evaluated. Treatment with anti-Nter domain and anti-H12 domain antibodies led to a marked reduction of ExoU secretion (Figure 1C, upper panel); nevertheless, a slight change in ExoU secretion was observed in the presence of the anti-H7 domain and anti-Cter domain antibodies, which implies that the Nter- and H12-specific antibodies provide better protection. Meanwhile, similar trend of the secretion of ExoU was observed when bacteria were grown in an ExoU-inducing medium (Figure 1C, lower panel). Furthermore, the effects of the anti-PcrV fragments antibodies on T3SS mediated cytotoxicity were accessed. As expected, both the four anti-PcrV subdomains antibodies showed significant inhibition of cytotoxicity (Figure 1D). And the activities of anti-Nter and -H12 antibodies were significant stronger than that of anti–H7 and –Cter antibodies, which was similar to the results from ExoU secretion assays. Collectively, these data indicate that Nter and H12 are immunodominant and more appropriate for vaccine development.

### Recombinant Nter-H12 (PcrV_NH_) Behaves as a Homogenous Monomer

**Figure 2:**
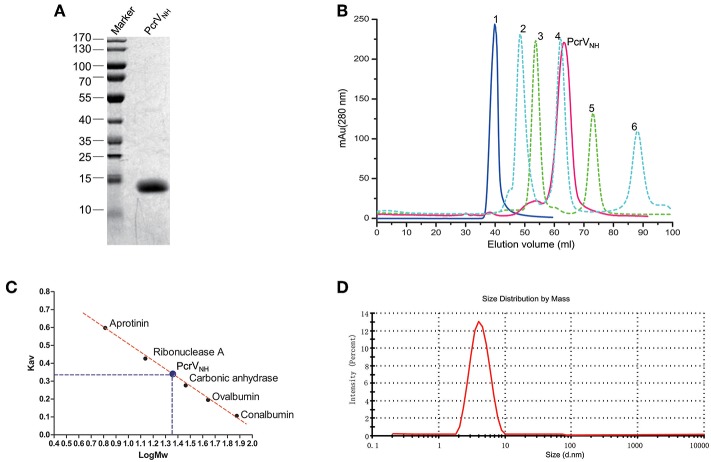
Characterization of recombinant PcrV_NH_. **(A)** SDS-PAGE analysis of PcrV_NH_. The purity of PcrV_NH_ was ~96% as determined by the density of the corresponding band on a SDS-PAGE gel. **(B)** The chromatograms curves of protein standards and PcrV_NH_ were overlaid for comparison. Curves 1 to 6 represent the peaks of standard proteins, namely, blue dextran 2000, conalbumin, ovalbumin, carbonic anhydrase, ribonuclease A, and aprotinin. The corresponding elution volume was 40.0, 47.3, 54.6, 62.1, 73.8, and 88.2 ml, respectively. PcrV_NH_ forms a symmetrical peak at 67.3 ml. **(C)** Linear regression of standard proteins to calculate the molecular weight of PcrV_NH_. The bar represents the Kav (gel phase-distribution coefficient) value of each protein. The standard curve was calculated as “Kav = 0.980–0.479*LogMw” (*R*^2^ = 0.996). The calculated Mw of PcrV_NH_ was ~21.2 KDa. **(D)** Dynamic light scattering analysis of PcrV_NH_ demonstrated that it forms a symmetrical peak with a diameter of 4.0 nm.

Building on the previous findings, we hypothesized that a combination of Nter and H12 domains could be a good vaccine candidate. After several attempts, we successfully generated an aqueous protein PcrV_NH_ (Figure 2A), which comprised Nter at the N terminus connected with the H12 domain by a Gly-Ser-Gly-Gly-Ser-Gly flexible linker (Figure 1A). Next, the oligomerization state and homogeneity of PcrV_NH_ were evaluated for their impact on antigen immunogenicity and industrial quality control. As shown in Figure 2B, PcrV_NH_ formed a symmetrical peak at 67.3 ml when eluted from the Superdex 200 size exclusion column. The elutions of standard protein blue dextran 2000, conalbumin, ovalbumin, carbonic anhydrase, ribonuclease A and aprotinin were 40.0, 47.3, 54.6, 62.1, 73.8, and 88.2 ml, respectively. Linear regression of Kav and Mw showed that the standard curve was calculated as “Kav = 0.980-0.479*LogMw” (*R*^2^ = 0.996). According to the formula, the calculated Mw of PcrV_NH_ was ~21.2 kDa (Figure 2C). In addition, PcrV_NH_ formed a single symmetrical peak, with a diameter of ~4.0 nm in dynamic light scattering analysis (Figure 2D). The predicted molecular weight based on the diameter was ~22.1 kDa, which suggests that PcrV_NH_ behaves as a monomer in solution because the theoretical molecular weight is ~21.2 kDa. These results combined indicate that PcrV_NH_ behaves as a homogeneous monomer in aqueous phase.

### Vaccination of PcrV_NH_ Induces Th1, Th2, and Th17 Responses

**Figure 3:**
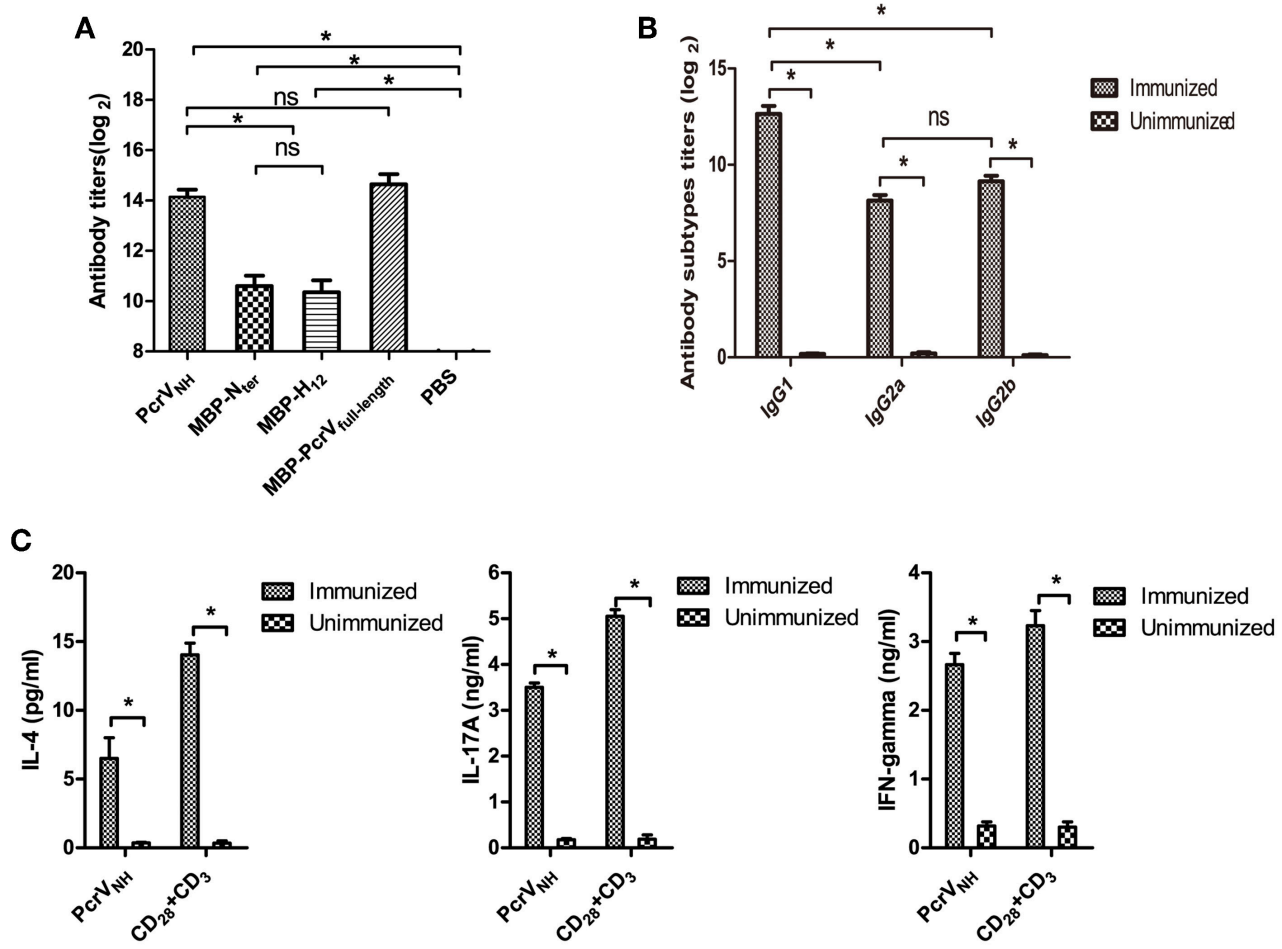
Analysis of PcrV_NH_-elicited immune responses. **(A)** PcrV_NH_-elicited humoral immune response. The bar represents the titer of IgG against PcrV_NH_, MBP-N_ter_, MBP-H_12_, and MBP-PcrV_full−length_ from PcrV_NH_-immunized mice **(B)**. The subtypes of anti-PcrV_NH_ antibodies. The bar represents the titer of IgG1, IgG2a, and IgG2b against PcrV_NH_ 7 days after the last immunization. **(C)** PcrV_NH_-elicited cellular immune response in the spleen. Splenocytes from PcrV_NH_-immunized mice were collected and stimulated with purified antigens. The bar represents the concentration of IL-4, IFN-γ, and IL-17A in the culture supernatant. The data are shown as the mean ± SE. Multiple comparisons among three groups were analyzed by one-way ANOVA. *P < 0.05, ns, no significance (*P* > 0.05).

To investigate the immunogenicity of recombinant PcrV_NH_, the levels of Nter-, H12- and PcrV_NH_- specific antibodies in PcrV_NH_ immunized mice were determined. As expected, the levels of anti-PcrV_NH_, anti-Nter, and anti-H12 antibodies were significantly elevated compared with those in the PBS group (*P* < 0.05) (Figure 3A). In addition, no significant differences in titer were observed between Nter- and H12-specific antibodies. Additionally, the PcrV_NH_ was able to react with MBP-PcrV_full−length_ at similar levels, which indicates the proper folding of the Nter and H12 domains in PcrV_NH_ (Figure 3A).

Next, the subtypes of IgGs induced by vaccination of PcrV_NH_ were determined. As expected, significantly increased levels of antigen-specific IgG1, IgG2a, and IgG2b were detected in PcrV_NH_-immunized mice (Figure 3B). In addition, the level of antigen-specific IgG1 was significantly higher than that of IgG2a or IgG2b, which suggests that PcrV_NH_ vaccination induces a Th2–predominant immune response. Furthermore, the levels of cellular immune responses in the spleen were investigated. Consistent with the IgG subtyping, the Th2–predominant immune response was also observed in the spleen based on a sharp increase of IL-4 levels upon stimulation with PcrV_NH_ (Figure 3C). Additionally, increased secretion of both IFN-γ and IL-17A was observed, suggesting an induction of Th1 and Th17 responses in the spleen.

### Vaccination of PcrV_NH_ Protects Mice in an Acute Pneumonia Model

**Figure 4:**
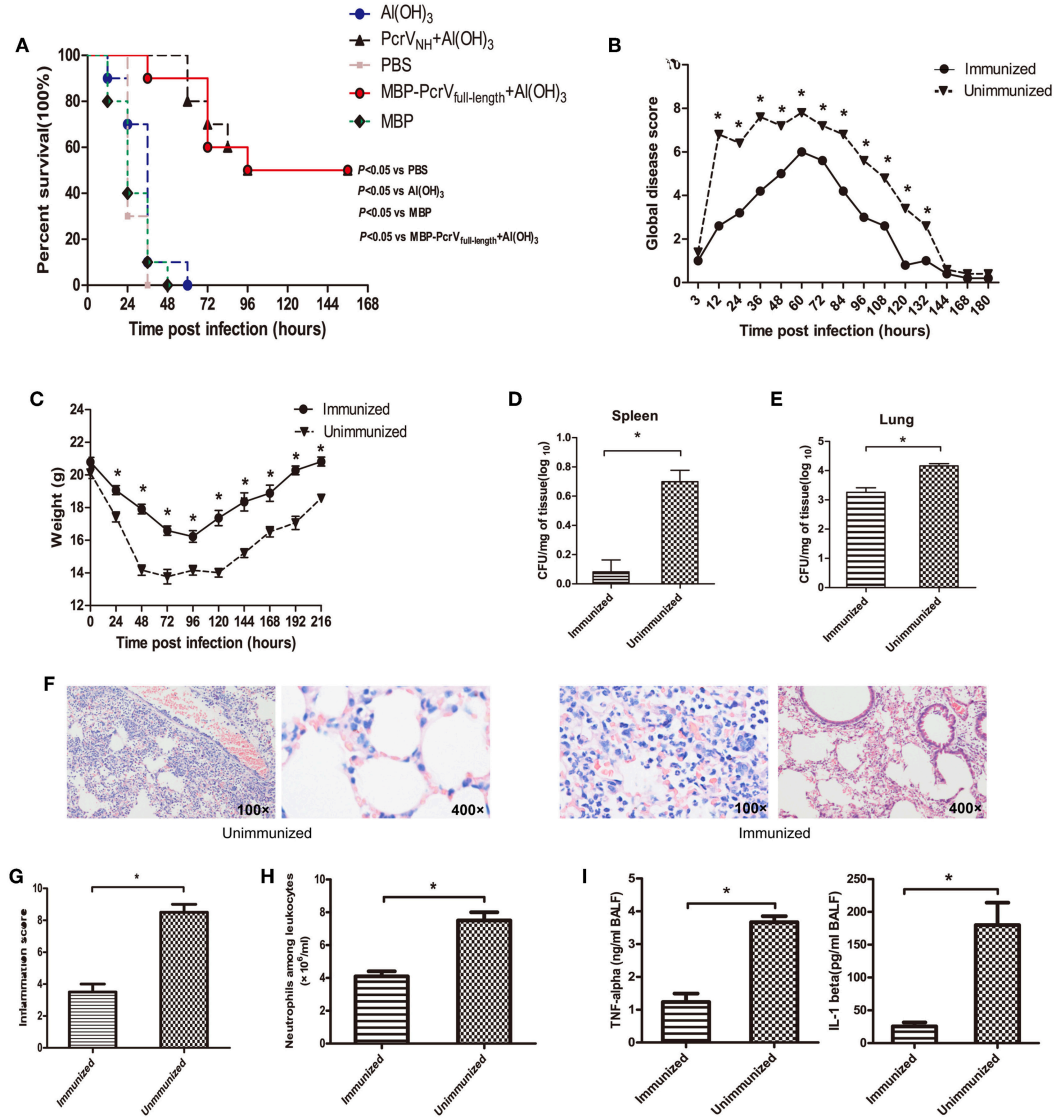
Vaccination of PcrV_NH_ confers protection in mice. **(A)** Mice (*n* = 10) were immunized with Al(OH)_3_, PcrV_NH_, PBS, MBP-PcrV_full−length_, or MBP. The survival of mice was recorded every 12 h after intratracheal injection with a lethal dose of PA XN-1 for 7 days. **(B)** The global disease score of mice immunized with PcrV_NH_ after challenge with a sublethal dose of PA XN-1 (*n* = 5). The score was recorded every 12 h for 8 days. **(C)** The weight loss of mice immunized with PcrV_NH_ after challenge with a sublethal dose of PA XN-1 (*n* = 5); the weight was recorded every 24 h for 9 days. **(D,E)** Assessment of the bacterial load in the lungs and spleen of PcrV_NH_-immunized mice 24 h after challenge with a sublethal dose of PA XN-1. The bar represents the log number of CFU per mg of spleen (left) and lung (right). **(F)** HE staining of lungs. PcrV_NH_-immunized mice were challenged with a sublethal dose of PA XN-1; 24 h later, the lung was collected and stained with hematoxylin and eosin. Images were captured at 100× magnification and 400× magnification. **(G)** Semiquantitative analysis of lung inflammation. The bar in the right panel represents the inflammation score. **(H)** Evaluation of neutrophil infiltration in the infected mice. The bars represent the number of neutrophils in 1 ml of BALF of immunized mice 24 h post-challenge. **(I)** Quantitative measurement of the pro-inflammatory cytokines TNF-α and IL-1β in the lungs. The data are shown as the means ± SE. *P < 0.05, ns, not significant (*P* > 0.05).

To determine whether vaccination of PcrV_NH_ is protective, immunized mice were challenged with a lethal dose of PA XN-1. As expected, a significant improvement in survival was observed in mice vaccinated with PcrV_NH_ compared with the PBS-, Al(OH)3-, and MBP- immunized mice (*P* < 0.05) (Figure 4A). Moreover, no significant differences in survival were observed between mice immunized with PcrV_NH_ and MBP-PcrV_full−length_ (*P* > 0.05), suggesting that PcrV_NH_-induced immunity is as effective as full-length PcrV. Similar trends of global disease and bodyweight changes were observed following a sublethal challenge of PA XN-1 (Figures 4B,C). To date, PcrV_NH_-immunized mice showed lower disease scores and loss of bodyweight than unimmunized mice. In addition, PcrV_NH_-immunized mice recovered within 120 h of challenge, which was earlier than that of unimmunized mice.

Next, PcrV_NH_-immunized mice were challenged with a sublethal dose of PA XN-1 to investigate the mechanism of PcrV_NH_-induced protection. First, the lungs and spleens of immunized were harvested 24 h post-infection, and their bacterial burden was evaluated. Notably, the vaccinated group showed significantly lower bacterial loads than the unvaccinated group (*P* < 0.05) (Figures 4D,E). Second, histological analysis of the lungs showed that mice from the PcrV_NH_ vaccination group exhibited less infiltration of inflammatory cells, bleeding, and tissue damage (Figure 4F). Consistent with the microscopy observations, the inflammation score of vaccinated mice was significant lower than that of the control group (Figure 4G) (*P* < 0.05).

Third, results of the quantitative analysis of neutrophil infiltration in BALF showed that the number of neutrophils from mice immunized with PcrV_NH_ was significantly lower than that of unimmunized mice (Figure 4H), which was consistent with the findings from the histological analysis. Finally, a significant reduction of pro-inflammatory cytokine (TNF-α and IL-1β) secretion was observed in immunized mice (Figure 4I). These results together indicate that PcrV_NH_-induced protection is mediated by reduction of bacterial burden, inflammation, and production of pro-inflammatory cytokines in the lung.

### Anti-PcrV_NH_ Antibodies Provide Protection Against PA Infection

**Figure 5:**
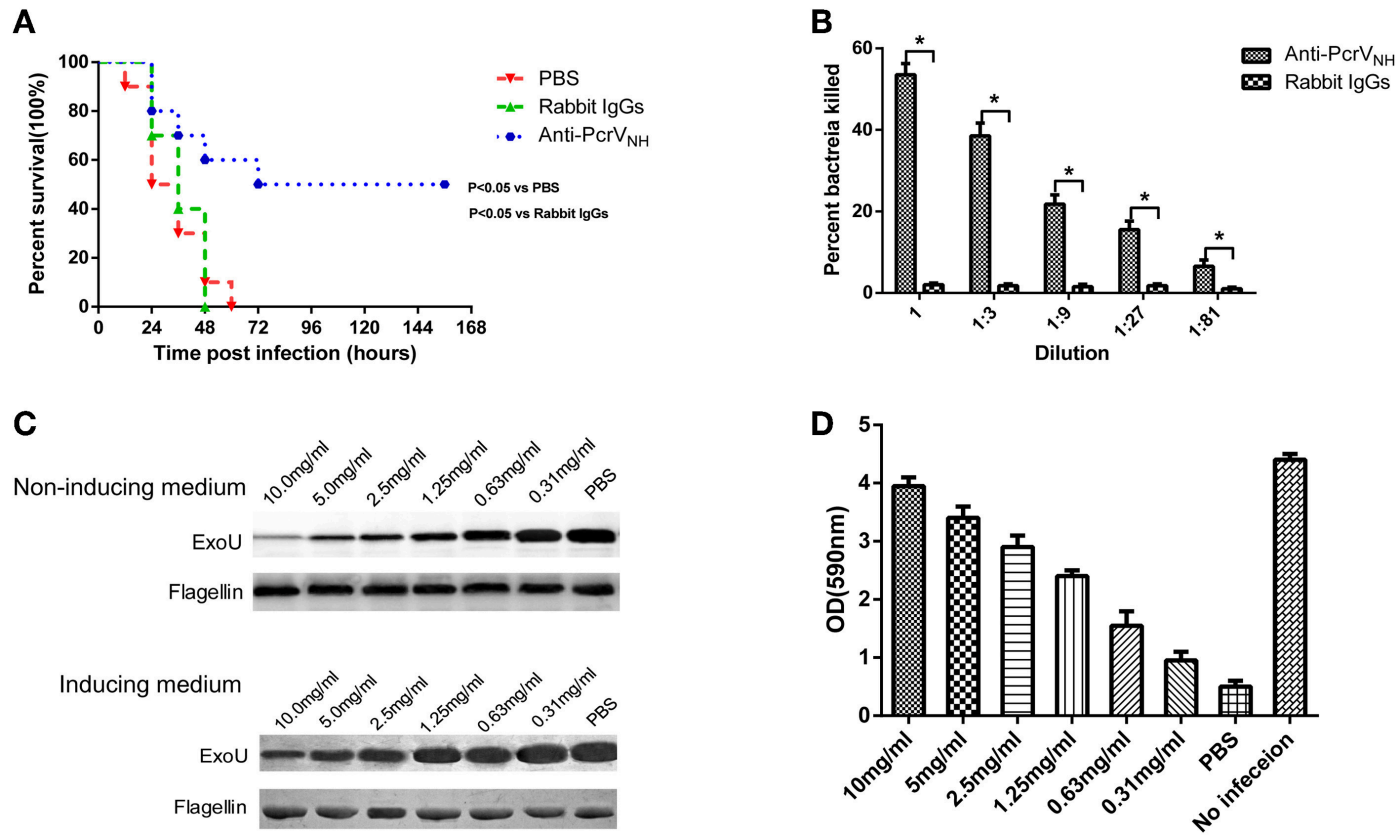
The protective efficacy of anti-PcrV_NH_ antibodies. **(A)** Passive immunization with anti-PcrV_NH_ antibodies protected mice challenged with a lethal dose of PA XN-1. Ten mice were intraperitoneally injected with 100 μl of anti-PcrV_NH_ IgG (25 mg/ml) 4 h before challenge. The survival of mice was recorded every 12 h for 7 days. **(B)** The opsonophagocytic killing activity of anti-PcrV_NH_ antibodies. Sera from immunized rabbits were diluted and incubated with PA XN-1. The bar represents the percentage of killed bacteria in a series of dilutions. The data are presented as the means ± SE. Anti-PcrV_NH_ antibodies showed marked bactericidal activity. *Significant difference between the anti-PcrV_NH_ group and the unimmunized group (*P* < 0.05). **(C)** The effect of anti-PcrV_NH_ antibodies on the secretion of ExoU. PA 103 grown in ExoU inducing and non-inducing media were co-incubated with a gradient (from 0.31 to 10 mg/ml) concentration of antibodies, and the ExoU in the culture supernatant was detected by Western blot. The content of flagellin in cell pellets was used as control. The anti-PcrV_NH_ antibodies were able to inhibit the secretion of ExoU in a dose-dependent manner. **(D)** The effect of anti-PcrV antibodies on the T3SS mediated cytotoxicity. Hela cells were infected with PA103 for 3 h and a gradient (from 0.31 to 10 mg/ml) concentration of anti-PcrV_NH_ antibodies was added into the culture media. The attached cells were stained crystal violet, which was then dissolved in ethanol. The bar represents the optical density at 590 nm of dissolved crystal violet. The T3SS mediated cytotoxicity reduced with the increase of the concentration of anti-PcrV_NH_ antibodies. The data are shown as the means ± SE.

In general, antigen-specific antibodies are responsible for vaccine-induced protection; thus, we investigated whether passive immunization of anti-PcrV_NH_ antibodies alone was protective. Ten mice were intraperitoneally injected with rabbit anti-PcrV_NH_ antibodies (1 mg/mouse) and then challenged with PA XN-1 intratracheally. Significantly prolonged survival was observed in mice given anti-PcrV_NH_, with a survival rate of 50%. However, no change in survival was found in mice immunized with non-specific rabbit IgGs (Figure 5A).

To further investigate the mechanism of anti-PcrV_NH_ antibody-mediated protection, an *in vitro* OPK assay was performed to determine the activity to induce antibody- and complement-mediated bacterial killing. As expected, in the presence of complement, anti-PcrV_NH_ antibodies exhibited significantly more potent opsonophagocytic activity in a concentration-dependent manner (Figure 5B). Nevertheless, no killing effect was observed from non-specific rabbit IgGs. In addition, the effect of anti-PcrV_NH_ antibodies on the secretion of ExoU was evaluated. The results showed that the amount of secreted ExoU in both inducing and non-inducing conditions were reduced with the increase in anti-PcrV_NH_ antibodies, while there was no change in non-specific rabbit IgGs at a concentration as high as 10 mg/ml (Figure 5C). In addition, the effects of anti-PcrV_NH_ antibodies on the T3SS mediated cytotoxicity were evaluated. As expected, a dose dependent inhibition of T3SS mediated cell detachment was observed (Figure 5D). Taken together, these results show that anti-PcrV_NH_ antibodies alone are able to provide protection against PA infection, probably by the induction of opsonophagocytosis and inhibition of ExoU secretion and T3SS mediated cytotoxicity.

### PcrV_NH_ Protects Against Multiple PA Clinical Isolates in Murine Pneumonia Models

**Figure 6:**
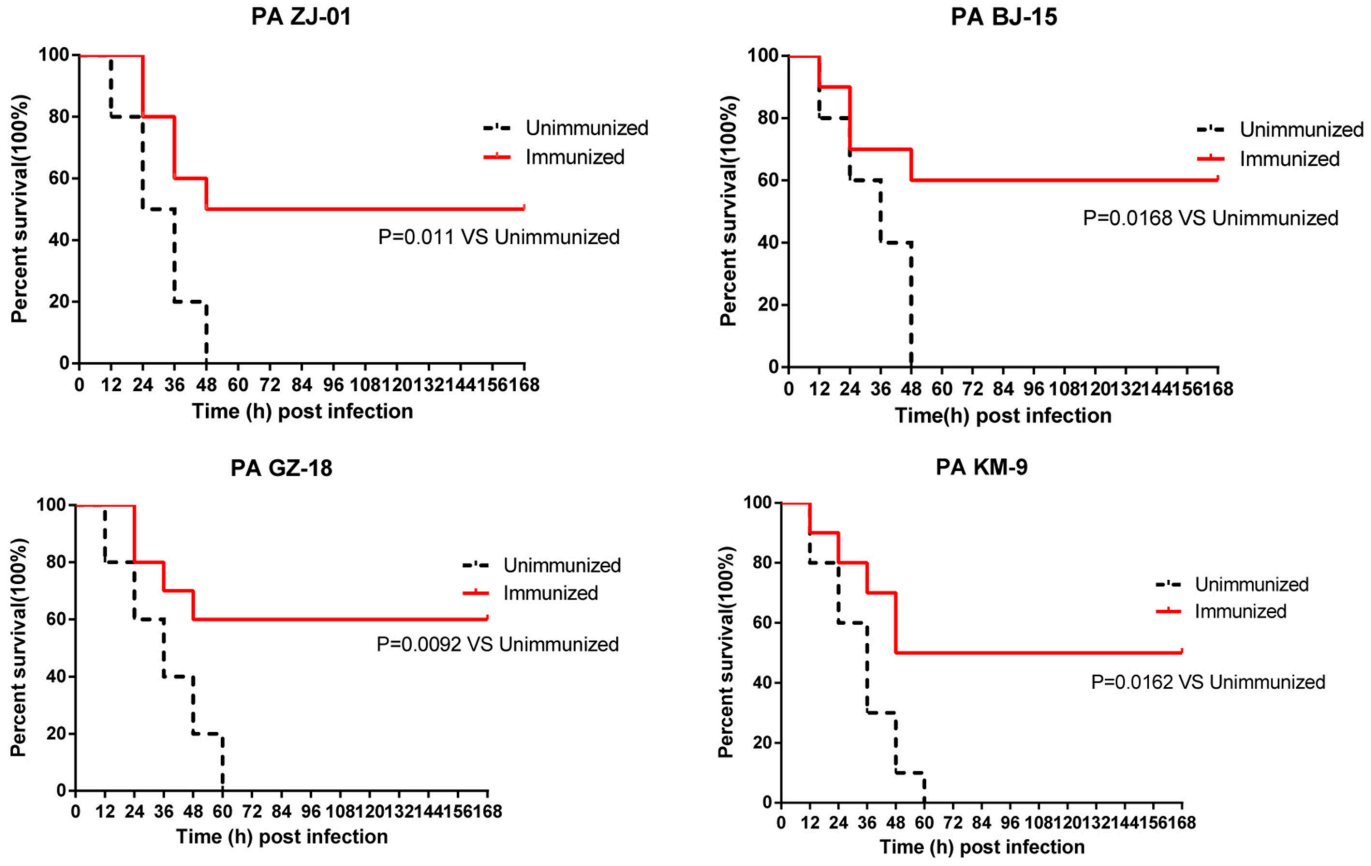
Immunization of PcrV_NH_ protects mice from challenge with a different PA clinical strain. Mice were challenged by intratracheal injection with lethal dose of PA ZJ-01, PA GZ-18, PA BJ-15, and PA KM-9. The dose of PA ZJ-01, PA GZ-18, PA BJ-15, and PA KM-9 was 3.0 × 10^7^ CFU, 2.0 × 10^7^ CFU, 2.0× 10^7^ CFU, and 4.0 × 10^6^ CFU, respectively. Survival rates were recorded every 12 h over 7 days.

To determine whether PcrV_NH_ could provide broad protection, mice immunized with PcrV_NH_ were challenged with four PA clinical strains isolated from hospitals in China, namely, PA ZJ-01, PA GZ-18, PA BJ-15, and PA KM-9. The serotypes of the four strains were O6, O11, O10, and O2, respectively, which represent the first four popular serotypes of PA from nosocomial pneumonia patients (30). Immunized mice had significantly better survival than unimmunized mice (Figure 6). In addition, the survival rate of immunized mice was 50, 60, 60, and 50% when challenged with PA ZJ-01, PA GZ-18, PA BJ-15, and PA KM-9, respectively. However, no unimmunized mice survived. Collectively, these data suggest that vaccination with PcrV_NH_ provides broad protection against multiple PA strains.

## Discussion

An increasing number of novel vaccine candidates are being designed with the help of protein structural biology tools (22). Antigens are rationally engineered to improve their solubility in water or to provide better protection (31, 32). Herein, we provide an additional approach for structure-based vaccine design. In brief, the structural information of the target antigen (PcrV in this case) was collected in advance and taken into consideration when designing the constructs. The purpose was to ensure the correct folding of each domain, which benefited their further applications. After screening the immunogenicity of each domain, the immunodominant domains were combined to generate an optimized vaccine candidate. This approach can also be used to optimize other important antigens.

Th1, Th2, and Th17 responses were simultaneously observed in mice vaccinated with PcrV_NH_ in our studies. Anti-PcrV_NH_ antibodies alone showed protective activities in *in vitro* and *in vivo*, and the Th2 response was therefore the leading pivotal factor for PcrV_NH_-mediated protection. Nevertheless, Th1 and Th17 cell-mediated immunity has emerged as an increasingly important factor in vaccine-induced protection. In the lung, Th17 cells mainly induced the recruitment of neutrophils to combat infection in an antibody-independent manner (33). In the PA-infected lung, protective IL-17 is constitutively produced at early stages. Moreover, recombinant PopB-PcrH and an OprL mutant elicited a strong Th17 response and conferred protection against multiple clinical PA isolates (28, 34). In this case, whether and how PcrV_NH_-induced Th17 responses contribute to protection need further investigations.

PcrV has been shown to be a good vaccine candidate in multiple animal models since it was first identified in 1999 (11, 17). However, it was not found in any PA vaccines in clinical trials. One possible explanation is that the full-length PcrV tended to precipitate in water, which hindered its further application in industry, as observed in our lab previously (21). In this study, we found that PcrV_NH_ was easy to produce and behaved as a homogenous monomer. In addition, PcrV_NH_-induced immunity was as effective as that of full-length PcrV. One possible reason could be that PcrV_NH_ is composed of the two immunodominant domains of full-length PcrV. Furthermore, the ELISA results demonstrated that their immunogenicity was properly preserved. Another explanation could be the introduction of the Gly-Ser-Gly-Gly-Ser-Gly linker in PcrV_NH_, which has been widely used for the generation of fusion proteins (35, 36). This flexible linker helped the Nter and H12 domains fold as in native PcrV.

Previously studies have showed that anti-PcrV antibodies mediated protections were Fc independent, as these antibodies were unable to activate complement-mediated killing (37). In addition, by a multilaser spinning-disk intravital microscopy, Thanabalasuriar et al. found that protection from anti-PcrV antibodies depend on the inhibition of T3SS cytotoxic activity and the acidification of endosomal compartments (14). Thus, it was interesting to note the opsonophagocytic killing activities of anti-PcrV_NH_ antibodies in our study, which suggests the participation of Fc domain in anti-PcrV provided protection. We deduce that there are three possible explanations for the *in vitro* opsonophagocytic activities of anti-PcrV_NH_ antibodies. Firstly, the recruitment of neutrophils is a hallmark of the response to PA infection, which kill bacteria by a number of highly effective molecules (38). Thus, neutrophilic HL-60 cells was applied in our study to ensuring the phagocytic killing, as widely used in other *in vitro* assays of opsonophagocytic activities of antibodies against PA (21, 39, 40). Secondly, anti-PcrV_NH_ antibodies may help the engulfment of PA by neutrophils, because an *in vivo* study have showed that a subtle increase of PA internalization when given anti-PcrV antibodies (14). Thirdly, anti-PcrV antibodies were able to increase the localization of ingested PA into acidified vacuoles (14), which results in an effective killing within the acidified phagosomes in neutrophils.

One important reason to select the Nter and H12 domains as a fusion subunit is the fact that antibodies against these two domains are able to block the PcrV-mediated secretion of ExoU. The effect of anti-H12 antibodies is not surprising because the H12 domain contributes to the oligomerization of PcrV to form the translocation channel (41). To date, the Nter domain has been considered a self-chaperone because of its sequential similarity with common chaperones; thus, little attention has been paid to this domain. Interestingly, anti-Nter antibodies showed effective inhibition of the secretion of ExoU in this study. Similarly, a nanobody, 13F07, which recognizes the Nter domain, was identified as a good biparatopic antibody candidate because of its functional blockade of PcrV (42). Collectively, these data emphasize an important role for the Nter domain of PcrV in the secretion of TTS effectors in addition to its roles as a self-chaperone.

One of the biggest challenges for the development of a PA vaccine is the variety of PA genomics. To date PA can be classified into almost twenty serotypes based on variations in the O antigen (43). A genomic study of PA clinical isolates showed that ~15% of non-redundant genes constitute the core genome (44), indicating a large number of variations among PA genomes. One strategy is to identify and test conserved proteins as vaccine candidates to elicit broad protection. Studies on 269 PA clinical isolates showed that ~87.7–90.2% of strains are capable of expressing PcrV (45–47). Thus, it is surprising to observe that PcrV_NH_ conferred effective protection against five clinical isolates with prevalent serotypes in our study.

In fact, PcrV_NH_ vaccine produced in this study induced at most 50% protection in acute PA pneumonia model, which was considerably lower than some PA vaccine candidates (48). One possible reason could be the application of aluminum hydroxide as adjuvant. In usual, aluminum hydroxide formulated antigens preferentially induced a type 2 immune response (49). Consistently, vigorous Th2 immune response was also observed after immunization with PcrV_NH_ formulated with aluminum. However, Th1 and Th17 responses, which play an increasingly important role in combating PA infection (50, 51), were not efficiently elicited. Another possible explanation could be that PA was able to cause infectious diseases by multiple pathogenic factors (38). Thus, it was not surprising to note the restricted protection by blocking of the PcrV or T3SS alone. For these reasons, PcrV_NH_ elicited protection could be enhanced by introduction of an optimized adjuvant, or by combination with other protective vaccine candidates.

In summary, the Nter and H12 domains of PcrV were determined as the two immunodominant domains with guidance from their structural information. Recombinant PcrV_NH_, which consists of Nter and H12, was produced in *E. coli* and behaved as a soluble homogenous monomer. Vaccination with PcrV_NH_ induced a multifactorial immune response and conferred effective protection against challenge with five PA clinical isolates. These data imply that PcrV_NH_ is a promising vaccine candidate for the control of PA infection.

## Ethics Statement

All animal care and experiments in this study abided by the ethical regulations and were approved by the Animal Ethical and Experimental Committee of the Third Military Medical University (NO. TMMU0159).

## Author Contributions

CW, JZ, LZ, and XC performed the major experiments. CW and QZ wrote the draft. CG and YW provided and analyzed the clinical samples. WX and QZ analyzed the data and revised the manuscript. JG designed the experiments and got the grants.

### Conflict of Interest Statement

The authors declare that the research was conducted in the absence of any commercial or financial relationships that could be construed as a potential conflict of interest.
